# Supplementary material for: Short noncoding RNAs as predictive biomarkers for the development from inflammatory bowel disease unclassified to Crohn’s disease or ulcerative colitis
Source: PLoS One. 2024 Feb 26;19(2):e0297353. doi: 10.1371/journal.pone.0297353 (PMC10896517; doi:10.1371/journal.pone.0297353)
Supplement: S1 File — (PDF) [file pone.0297353.s001.pdf]

Supplementary file 1

Name of the primers	Sequence
hsa-miR-182-5p_Foward_2	Gtttggcaatggtagaactca
hsa-miR-182-5p_Reverse_3	Ggtccagtttttttttttttttagtgt
hsa-miR-451a_Forward_4	Cgcagaaaccgttacca
hsa-miR-451a_Reverse_2	Gtccagtttttttttttttttaactcag
hsa-miR-628-5p_Forward_2	Cgcagatgctgacatatttac
hsa-miR-628-5p_Reverse_3	Ggtccagtttttttttttttttcct
hsa-miR-671-5p_Forward_3	Gccctggaggggct
hsa-miR-671-5p_Reverse_2	Ggtccagtttttttttttttttcct
hsa-miR-1298-3p_Forward_3	Gcatctgggcaactgact
hsa-miR-1298-3p_Reverse_4	Ggtccagtttttttttttttttagttc
hsa-miR-4793-3p_Forward_4	Gcagtctgcactgtgagt
hsa-miR-4793-3p_Reverse_1	Gtccagtttttttttttttttagcca
ENSG00000239080_Left	GAGATGTGCCACCCTTGAAC
ENSG00000239080_Right	ATGGGTAAGATGCCAACGTC
hsa-MiR-27a-3p_Forward_1	Gggcttagctgcttgtg
hsa-MiR-27a-3p_Reverse_2	Gtccagtttttttttttttttgctc
hsa-miR-16-5p_Forward_2	Cgcagtagcagcacgta
hsa-miR-16-5p_Reverse_1	Cagtttttttttttttttcgccaa
hsa-miR-191-5p_Forward_1	caacggaatcccaaaagca
hsa-miR-191-5p_Reverse_5	tccagtttttttttttttttcagct
